# Deficits in the pitch sensitivity of cochlear-implanted children speaking English or Mandarin

**DOI:** 10.3389/fnins.2014.00282

**Published:** 2014-09-09

**Authors:** Mickael L. D. Deroche, Hui-Ping Lu, Charles J. Limb, Yung-Song Lin, Monita Chatterjee

**Affiliations:** ^1^Department of Otolaryngology, Johns Hopkins University School of MedicineBaltimore, MD, USA; ^2^Department of Otolaryngology - Head and Neck Surgery, Chi-Mei Medical Center, Taipei Medical UniversityTainan, Taiwan, China; ^3^Auditory Prostheses and Perception Laboratory, Boys Town National Research HospitalOmaha, NE, USA

**Keywords:** pitch, cochlear implants, plasticity, auditory development, tonal language

## Abstract

Sensitivity to complex pitch is notoriously poor in adults with cochlear implants (CIs), but it is unclear whether this is true for children with CIs. Many are implanted today at a very young age, and factors related to brain plasticity (age at implantation, duration of CI experience, and speaking a tonal language) might have strong influences on pitch sensitivity. School-aged children participated, speaking English or Mandarin, having normal hearing (NH) or wearing a CI, using their clinically assigned settings with envelope-based coding strategies. Percent correct was measured in three-interval three-alternative forced choice tasks, for the discrimination of fundamental frequency (F0) of broadband harmonic complexes, and for the discrimination of sinusoidal amplitude modulation rate (AMR) of broadband noise, with reference frequencies at 100 and 200 Hz to focus on voice pitch processing. Data were fitted using a maximum-likelihood technique. CI children displayed higher thresholds and shallower slopes than NH children in F0 discrimination, regardless of linguistic background. Thresholds and slopes were more similar between NH and CI children in AMR discrimination. Once the effect of chronological age was extracted from the variance, the aforementioned factors related to brain plasticity did not contribute significantly to the CI children's sensitivity to pitch. Unless different strategies attempt to encode fine structure information, potential benefits of plasticity may be missed.

## Introduction

A fine sensitivity to pitch is desirable not only for music perception (McDermott and Oxenham, 2008) but also for many aspects of speech perception, including the perception of prosody, speaker identity, voice emotion, and the separation of target speech from a noisy background in auditory scene analysis (respectively, Lehiste, 1970; Bregman, 1990; Murray and Arnott, 1993; Cutler et al., 1997; Bird and Darwin, 1998; Hillenbrand and Clark, 2009). In tonal languages, the listener must analyze pitch changes at both the syllabic and sentence levels to decode the semantic meaning (lexical tones) and the communicative intent or mood of the speaker. This places even greater informational emphasis on voice-pitch processing (Chao, 1968; Howie, 1976; Liu and Pell, 2012). In normal hearing (NH), the narrow auditory filters in the low frequency/apical region of the cochlea resolve the broadband harmonic complex into its constituent spectral peaks; the place-specific periodicity cues contribute to salient pitch sensation and fine pitch discrimination (Meddis and O'Mard, 1997; Bernstein and Oxenham, 2005). At the same time, the broad high frequency auditory filters integrate multiple harmonics into their response and their outputs preserve periodicity in the temporal envelope, resulting in unresolved pitch (Houtsma and Smurzinski, 1990; Meddis and O'Mard, 1997). Cochlear implants (CIs), although unable to transmit the resolved spectro-temporal fine structure needed to support a strong pitch sensation, do transmit the temporal envelope pitch to the extent allowed by the low-pass filter of the envelope extraction process. Unfortunately, the temporal envelope pitch does not result in a salient pitch percept in either normally hearing or implanted listeners (Shannon, 1983; Zeng, 2002; Chatterjee and Peng, 2008; Kong et al., 2009; Kong and Carlyon, 2010), thus limiting all of the aforementioned auditory skills for which a fine sensitivity to pitch is necessary (Gfeller et al., 2000; Leal et al., 2003; Kong et al., 2004, 2005; Fu et al., 2005; Luo et al., 2007; Stickney et al., 2007; Chatterjee and Peng, 2008; Peng et al., 2009). For young children, pitch changes acquire added importance, as, across cultures, the exaggerated prosody of infant-directed speech is thought to play a key role in language acquisition (Fernald et al., 1989; Bornstein et al., 1992; Jusczyk et al., 1992; Jusczyk, 1999; Soderstrom et al., 2003; Thiessen et al., 2005).

Today many children are implanted at a very young age, and yet, except for a few studies (e.g., Barry et al., 2002; Kopelovich et al., 2010), most of the literature on CIs and pitch perception has targeted either post-lingually deaf adults who learned to hear and speak with a functioning auditory periphery, or pre-lingually deaf adults who had little auditory input growing up and received a CI relatively late in life. In the present study, we investigated pitch processing by school-aged children with CIs. Most of these children were implanted relatively early in life, and acquired their native language through the CI. We hypothesized that the drive to acquire language and communicate in young children, together with the greater neural plasticity during the early years, might place them at an advantage relative to their later-implanted counterparts in pitch discrimination. Specifically, we wondered if these young children might be better able to extract pitch information from the limited cues available through the device. In addition, we hypothesized that the advantage might be even greater for children growing up in a tonal-language environment. Further, we were interested in effects of developmental age and duration of experience with the device. To test these hypotheses, pitch discrimination was measured for harmonic complexes and amplitude-modulated noise stimuli in a standard procedure and compared between CI children in the US and Taiwan and their normally-hearing (NH) peers, as a function of chronological age, age at implantation, duration of CI experience, and native language. Both kinds of stimuli were used to provide insight into how CI children process voice pitch in everyday life, and how NH children compare with CI children in their processing of temporal-envelope-based pitch. A child-friendly interface had been developed by Deroche et al. (2012) to collect data on the same tasks by English-speaking NH children, listening through headphones. The same interface was used here, except that sounds were played through loudspeakers instead, for a fairer comparison with implanted children. From the results of the previous study, it was expected that NH children would display a fine sensitivity to the fundamental frequency (F0) of harmonic complexes and a poor sensitivity to the amplitude modulation rate (AMR) of noise stimuli, keeping in mind that a large variability might exist between individuals. For CI children, deficits in F0 sensitivity were generally expected given the type of pitch cues they receive. However, it was hypothesized that those children who were implanted earliest, and/or had extensive experience with their implant, and/or were developing in a tonal language environment, might be relative experts at discriminating changes in temporal envelope modulations.

## General methods

### Listeners

Four groups of listeners participated. In the US, 34 normally-hearing (NH_US) and 47 cochlear-implanted (CI_US) children, all native speakers of English, were tested. In Taiwan, 11 normally-hearing (NH_TN) and 24 cochlear-implanted (CI_TN) children, all native speakers of Mandarin, were tested. The chronological age of children ranged from 4.6 to 21.3 years; their age at implantation varied from 10 months to 12 years of age; and duration of CI experience varied from 4 months to 17 years (details in Table 1). Age at profound hearing loss varied depending on the etiology: a majority of children (60%) were either deaf from birth or lost their hearing throughout their first year of life while a minority (5 out of the 71 implanted children) lost their hearing between 4 and 8 years of age. CI_TN children were all unilaterally implanted, while 55% of the CI_US children were implanted on both sides. The 26 children implanted bilaterally were tested only on the side implanted first. When there was any chance that the child could hear from the contralateral ear, because of a second implant, a hearing aid, or some residual hearing, the implant or hearing aid was removed and the contralateral ear was plugged with ear-foam.

**Table 1:** **Demographics of the four groups of listeners**.

	**Chronological age mean (*SD*) (min–max)**	**Age at implantation mean (*SD*) (min–max)**	**Duration of CI experience mean (*SD*) (min–max)**	**Age at profound hearing loss mean (*SD*) (min–max)**
NH_US	10.7 (3.1) (6.2–19.0)			
NH_TN	10.1 (3.4) (4.7–15.6)			
CI_US	11.8 (3.4) (6.4–18.4)	3.6 (3.0) (1.0–12.0)	8.2 (4.1) (0.3–16.9)	1.2 (2.0) (0.0–8.0)
CI_TN	13.2 (4.3) (6.5–21.3)	2.9 (1.2) (0.8–5.3)	10.4 (3.9) (2.9–17.0)	1.1 (0.7) (0.1–2.7)

Among the CI_US children, twenty-three had a *Cochlear* device: fifteen wore a Nucleus 24, four wore a Nucleus Freedom, and four wore a Nucleus CI512, all using the ACE speech processing strategy. Twenty-three others had an *Advanced Bionics* device: ten wore a Clarion CII and thirteen wore a Clarion HiRes90k, with different processing strategies (10 HiRes, 8 Fidelity-120, 3 CIS, 1 SAS, and 1 MPS). One child had a *Med-El* device C40+, using the HD-CIS processing strategy. Among the CI_TN children, nineteen had a *Cochlear* device: fifteen wore a Nucleus 24 using the ACE processing strategy, and 4 wore a Nucleus 22 using the ACE or the SPEAK strategy. Five others had *Med-El* devices: two wore a C40+ using the CIS strategy; two wore a Sonata with a FSP strategy, and one wore a Pulsar with a FSP strategy. On the whole, stimulation strategies were therefore primarily envelope-based. All children used their clinically assigned settings.

### Stimuli

There were four tasks: F0 discrimination at reference F0s of 100 and 200 Hz and AMR discrimination at reference rates of 100 and 200 Hz. The F0 tasks used harmonic complexes with partials up to the Nyquist frequency, all in sine phase and with equal amplitude. The AMR tasks used broadband Gaussian white noise, different for each of the three intervals, and freshly generated in each trial, modulated sinusoidally at a given rate with 100% depth. All stimuli were 300-ms long and gated by 10-ms ramps consisting in half a period of a raised cosine. The inter-stimulus duration was identical to the stimuli duration. The level of each stimulus, regardless of its AMR or its F0, was first equalized at 65 dB SPL and presented with a ±3 dB rove, to discourage listeners from using loudness cues. Note, however, that the ±3 dB rove applied at the acoustic input would translate to different degrees of rove of electrical current for implanted children, depending on their settings.

### Protocol

The rationale for the study and the protocol were first explained carefully to the children. There was no need for sign language interpreter because all implanted children had sufficiently good speech understanding. The experimenter reinforced the idea that the focus was on pitch and not loudness, since conceptualization and labeling of these percepts is often confused in the pediatric population (Andrews and Deihl, 1970; Hair, 1981). After obtaining consent from both children and parents, the children were invited to seat in the auditory booth and started the practice blocks. Each task followed a 3-interval 3-alternative forced choice (3I-3AFC) procedure: a listener always heard three intervals, two with the same F0/AMR (the reference) and the target interval with a higher F0/AMR. The target interval was placed with equal probability in the first, second or third interval. The listener was asked to report which interval sounded *different* in pitch (although it was always *higher* in pitch). Practice blocks differed from test blocks in several respects: (a) trials were presented at a slower pace as stimuli were 500-ms long and separated by 500-ms inter-stimulus intervals; (b) stimuli were presented without level roving; and (c) there were only 10 trials measuring performance for a fixed difference in F0/AMR. The purpose of practice was not only to familiarize listeners with the task, but also to optimize data collection during test in a range of F0/AMR differences that was neither at floor nor at ceiling. This step is particularly important since pitch sensitivity varies logarithmically depending on the nature of the underlying cues. For the NH population, there were strong expectations according to the previous study (Deroche et al., 2012) that a difference in F0 of 2 semitones or a difference in AMR of 1 octave represented easily discriminable percepts. For the CI population, it was much less obvious what target F0/AMR should be used to train children on. Some implanted children performed well with differences as small as 2 semitones in which case test sessions started rapidly after a couple of practice blocks. Other implanted children with poorer sensitivity were probed with increasingly larger differences in F0/AMR. Given the variability between blocks, it could take up to 15 blocks (i.e., 150 trials) to find a value at which performance was reliably above chance. When performance remained poor by a difference of 2 octaves, which occurred mostly among CI listeners or for the AMR task, the task was abandoned as performance would presumably not have improved with higher target rates. For both NH listeners (Burns and Viemeister, 1976) and CI listeners (e.g., Zeng, 2002), temporal pitch is unlikely discriminable above 400–800 Hz, i.e., 2 octaves above the reference rates used here.

A test block consisted of 70 trials (seven increments in F0 or AMR, tested 10 times each), presented in random order. The seven conditions were constructed by linear steps between 0.01 semitone and Δmax, the largest difference in F0/AMR chosen by the experimenter in view of the performance reached in the practice blocks. For example, when a child performed well, e.g., at 80% in a couple of practice blocks for a difference of one octave, the seven experimental conditions corresponded to differences of 0.01, 2, 4, 6, 8, 10, and 12 semitones. A difference of 1 cent (0.01 semitone) was used instead of no difference at all simply to avoid a forced-choice situation which has technically no correct answer; performance was nonetheless expected to be at chance level in this condition. Listeners completed between 1 and 6 test blocks (70–420 trials) for a given task, depending on the children's ability, their willingness to participate, and the variability in performance across blocks. When performance was very stable from one block to the next and increased gradually from chance to ceiling across the seven increments in F0/AMR tested, threshold could be located with accuracy with only 2 or 3 blocks. However, this ideal case scenario did not always occur. On one hand, the introduction of the level roving was disruptive for some children who had poorer performance at Δmax than was previously measured during practice. When this happened, the experimenter chose a larger Δmax and ran a new test block on an easier set of conditions. On the other hand, listeners (especially NH) generally improved over the course of several test blocks. Depending on the strength of this perceptual learning, performance could rise close to ceiling for most conditions (except the 1-cent difference), leaving few or no data points between floor and ceiling. When this happened, the experimenter chose a lower Δmax and ran a new test block on a more challenging set of conditions.

The interface was described in detail in Deroche et al. (2012): a cartoon became animated over the auditory presentation of each interval in synchronization with the visual presentation of a button. At the end of the third interval, the three buttons and three cartoons reappeared on the screen and the child clicked on the button for which the sound was perceived to be different. Response time (RT) was recorded for each trial from the instant the three buttons reappeared on the screen to the instant the listener provided a response. Listeners were not instructed to respond fast, but simply to be as accurate as possible in a timely manner. Feedback was provided via smiley faces (happy, excited, sad, or disappointed) and some winning/losing points. The experimenter provided verbal support and encouragement to boost the child's motivation and attention. A typical experimental session lasted 1.5–2 h: the youngest or more distracted listeners could not attend for more than an hour-long session at a time (with breaks), while the oldest and focused listeners were able to remain focused for 3-h long sessions (with breaks). Listeners were paid for their participation. Protocols were approved by the Institutional Review Board of the three different institutions at which this study took place.

### Equipment and testing sites

The study took place in three different research facilities. Data for the 35 Taiwanese children were collected using the same equipment at two sites in Taiwan: the Chimei Medical Center in Tainan and at the Chang Gung Memorial Hospital in Taoyuan. Data for all NH_US children and 9 CI_US children were collected at the Auditory Prostheses and Perception Laboratory of Boys Town National Research Hospital (BTNRH) in Omaha (US). Data for 38 CI_US children were collected at the Music Perception Laboratory of Johns Hopkins Hospital in Baltimore (US). Despite some discrepancies in experimental setups, signals were always sampled at 44.1 kHz and 16-bit resolution, presented via an external soundcard (Edirol UA at sites in the US; Soundmax Integrated Digital HD Audio in Taiwan) and a single loudspeaker, located approximately 2 feet from the child, at an average level of 65 dB SPL. Loudspeakers (Sony SS-MB150H at Johns Hopkins, Grason Stadler GSI at BTNRH; SB-1 Audio Pro in Taiwan) were placed directly facing the child, and a user-interface was displayed on a monitor located inside the booth. Listeners provided their responses using a touch-screen, a keyboard, a joystick, or a mouse, depending on the available equipment and the child's preference. Different auditory booths were used in the different sites, so their dimensions and reverberation characteristics varied. They were nonetheless all used for clinical or research purposes and had typical audiometric qualities: walls were sound absorbing and isolating from external sound. It was therefore unlikely that reverberation in any of the booths was sufficient to impede the depth of envelope modulations significantly. Furthermore, simple *t*-tests revealed no difference in performance between the different sites within the NH population, and within the CI population, suggesting that the different amounts of reverberation offered in the different booths had little influence on temporal pitch perception.

## Data analysis

### Trials with long RTs

Adult listeners retain a reasonably accurate memory of the pitch of pure tones up to 15 or 16 s after presentation (Bachem, 1954; Ross et al., 2004). While similar data are not available for children or for broadband harmonic complexes, responses provided more than 16 s after stimulus presentation were assumed to be unreliable, and those data were discarded prior to analyses.

### Overall performance different from chance

In many instances, children showed very poor performance during test (with level roving) while they reached high scores during the practice blocks (without level roving). These cases occurred more often for the AMR tasks, particularly at 200 Hz, the most difficult task. When this happened, the data collected during the test blocks were extremely noisy, not necessarily monotonic and the maximum likelihood technique (next section) could not deliver any acceptable fit. It was therefore useful to determine whether overall performance data for a given task was different from chance. A simple *t*-test was used at a significance level of 0.05. When this *t*-test was not significant, the data were judged to be at chance and shown in the results outside of the scale of the respective psychophysical parameter.

### Maximum likelihood technique

Performance data were fitted using the maximum-likelihood technique described by Wichmann and Hill (2001a,b). This technique is particularly powerful in that it gives more weight to the performance data which have been measured over a larger number of trials. It was consequently very well suited to the present data that were collected over several test blocks on possibly different sizes of F0/AMR difference. Chance level fixed the lower asymptote of the Weibull function defined below:

$$\Psi \left(x;\alpha ,\beta ,\lambda \right)=\frac{1}{3}+\left(\frac{2}{3}-\lambda \right)\left(1-exp^{-{\left(\frac{x}{\alpha }\right)}^{\beta }}\right)\;0\leq x<\infty$$

where Ψ is the percent correct score, *x* the difference of F0/AMR in semitones, α and β the parameters influencing the shape of the Weibull function and λ the lapse rate. Lapse rate is usually taken as evidence of inattention errors, and is often constrained in the range of 0–10%. In the present study, however, it reflected more generally the difficulty of a given task. Some listeners had extreme difficulties performing a task or could not perform above chance with the largest difference in F0/AMR. Lapse rate could thus be better appreciated in terms of the highest level of performance that could ever be achieved with the most discriminable target, and was therefore completely unconstrained, i.e., up to possibly 60%. In most cases, however, when lapse rate was in the range of 50–60%, the full data set was not significantly different from chance, and therefore a fit was not attempted. Discrimination threshold was extracted at half way between the lower and upper asymptotes and the slope was defined as the gradient of the Weibull function at threshold and expressed as % per unit, the unit depending on the value of the lapse rate (see Section Standardize Thresholds and Rescale Slopes).

**Figure 1:**
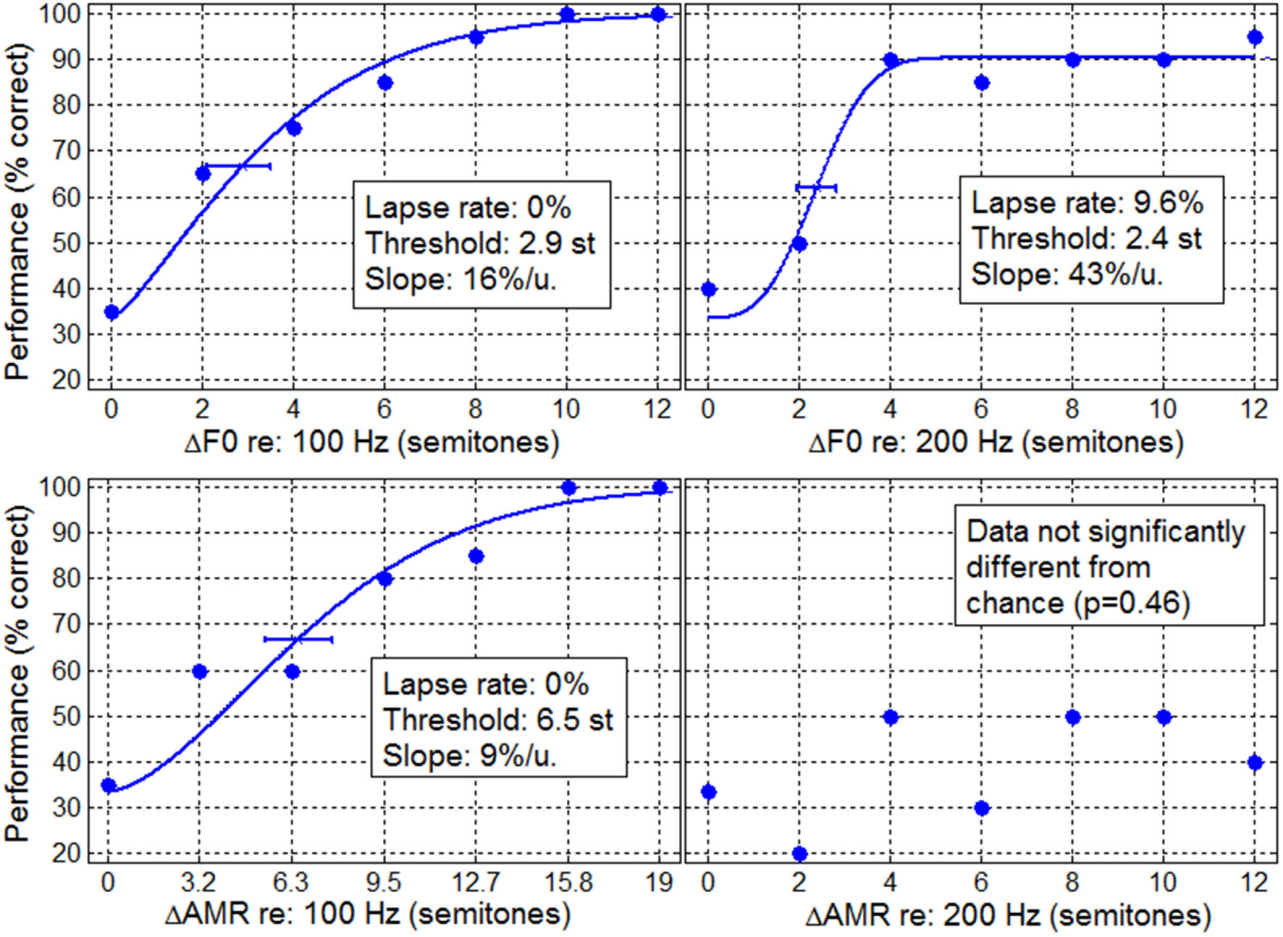
**Typical example of performance data for an implanted child in the F0 discrimination task (top panels) and in the AMR discrimination task (bottom panels) at 100 Hz (left) and 200 Hz (right)**. Lines represent the psychometric fits obtained from the maximum likelihood technique from which lapse rate, threshold, and slope were extracted.

A typical example is shown in Figure 1, for an implanted child from the CI_US group, who was 9.3 year old, implanted at 1.4 year old, and had his implant for 7.9 years. Performance data were above chance for the two F0 tasks as well as the AMR task at 100 Hz, providing reliable materials to fit psychometric functions and to obtain relatively accurate estimates of lapse rate, threshold, and slope. In contrast, this child could not perform above chance in the AMR task at 200 Hz. Attempting to fit a psychometric function on these very noisy data (AMR at 200 Hz) would lead to very inaccurate estimates of threshold and slope.

### Standardize thresholds and rescale slopes

Depending on the value of the lapse rate, threshold corresponded to different levels of performance. For instance, at a 0% lapse rate, threshold corresponded to a performance of 66.6%, equivalent to a *d*′ of 1.12 in signal detection theory for a 3I-3AFC procedure. At a 30% lapse rate, threshold corresponded to a performance of 51.6%, equivalent to a *d*′ of 0.61 for the same procedure. To provide a fairer comparison between subjects and between conditions, threshold was assumed to be proportional to *d*′, and all thresholds were thus standardized at the same *d*′ of 0.77, a value corresponding to a performance of 70.7% in a 2I-2AFC task. For example, a threshold of 3 semitones at a *d*′ of 1 was standardized to 2.31 semitones at a *d*′ of 0.77.

The slope, extracted from the gradient of the Weibull at threshold has a unit normalized by the scale between the lower and upper asymptote, i.e., different for different listeners. Slope estimates were thus rescaled by multiplying the Weibull gradient by (2/3-λ), such that slopes could be expressed in % per semitone for all listeners and conditions.

## Results

**Figure 2:**
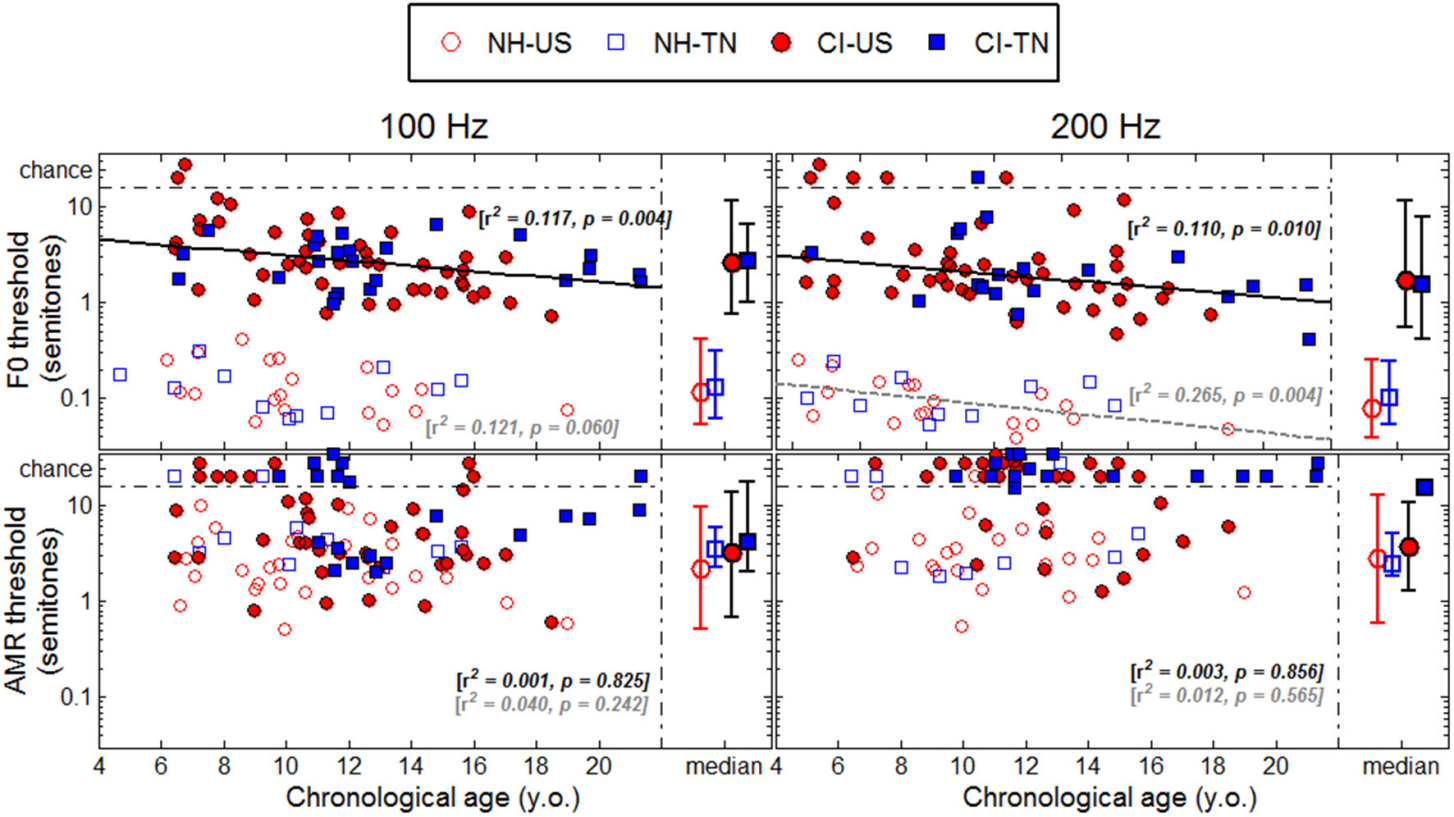
**Thresholds (standardized at a *d*′-value of 0.77) as a function of chronological age**. The medians are represented on the right side of each panel with the confidence intervals at 95% in each population.

**Figure 3:**
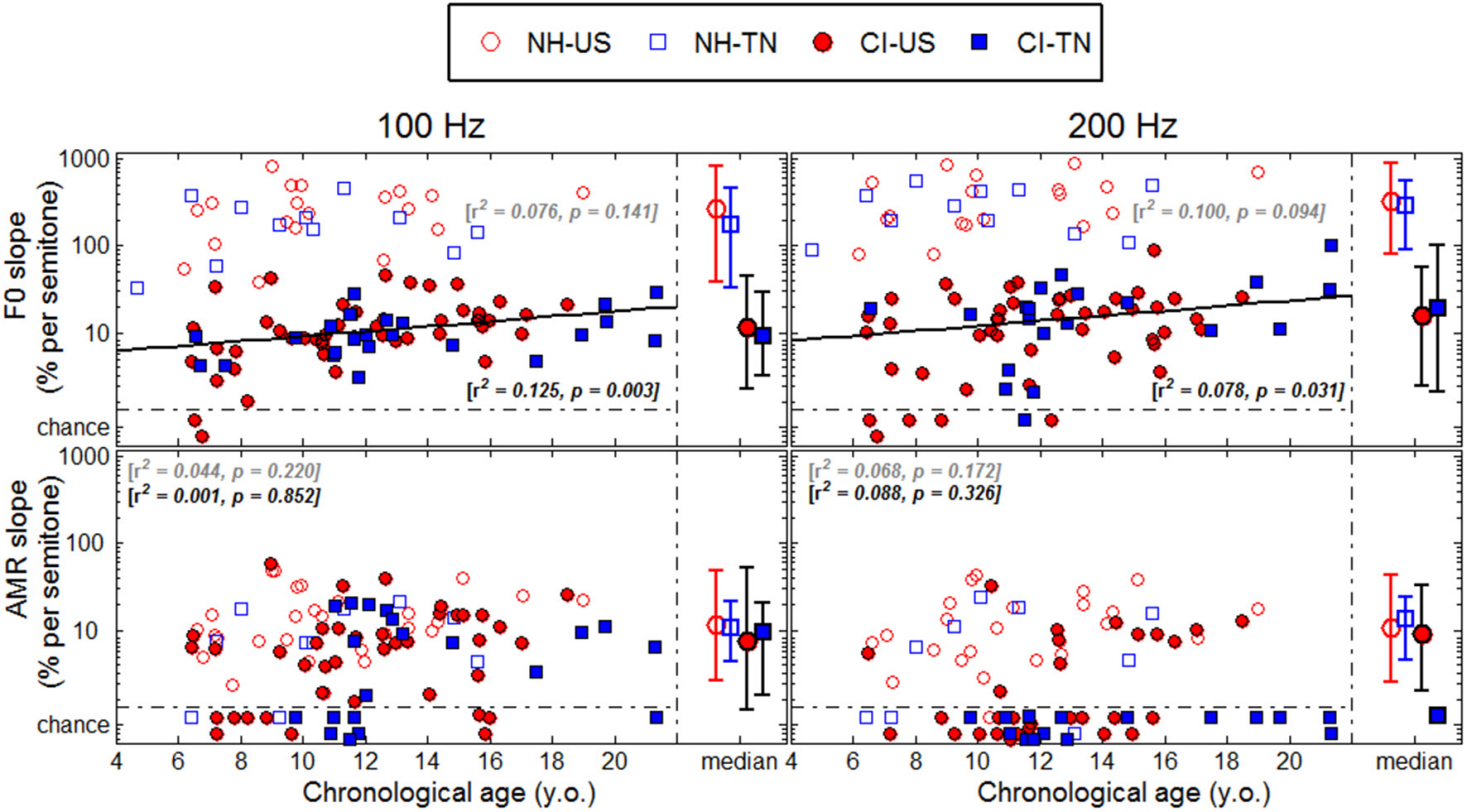
**Same as Figure 2 for slopes**.

**Figure 4:**
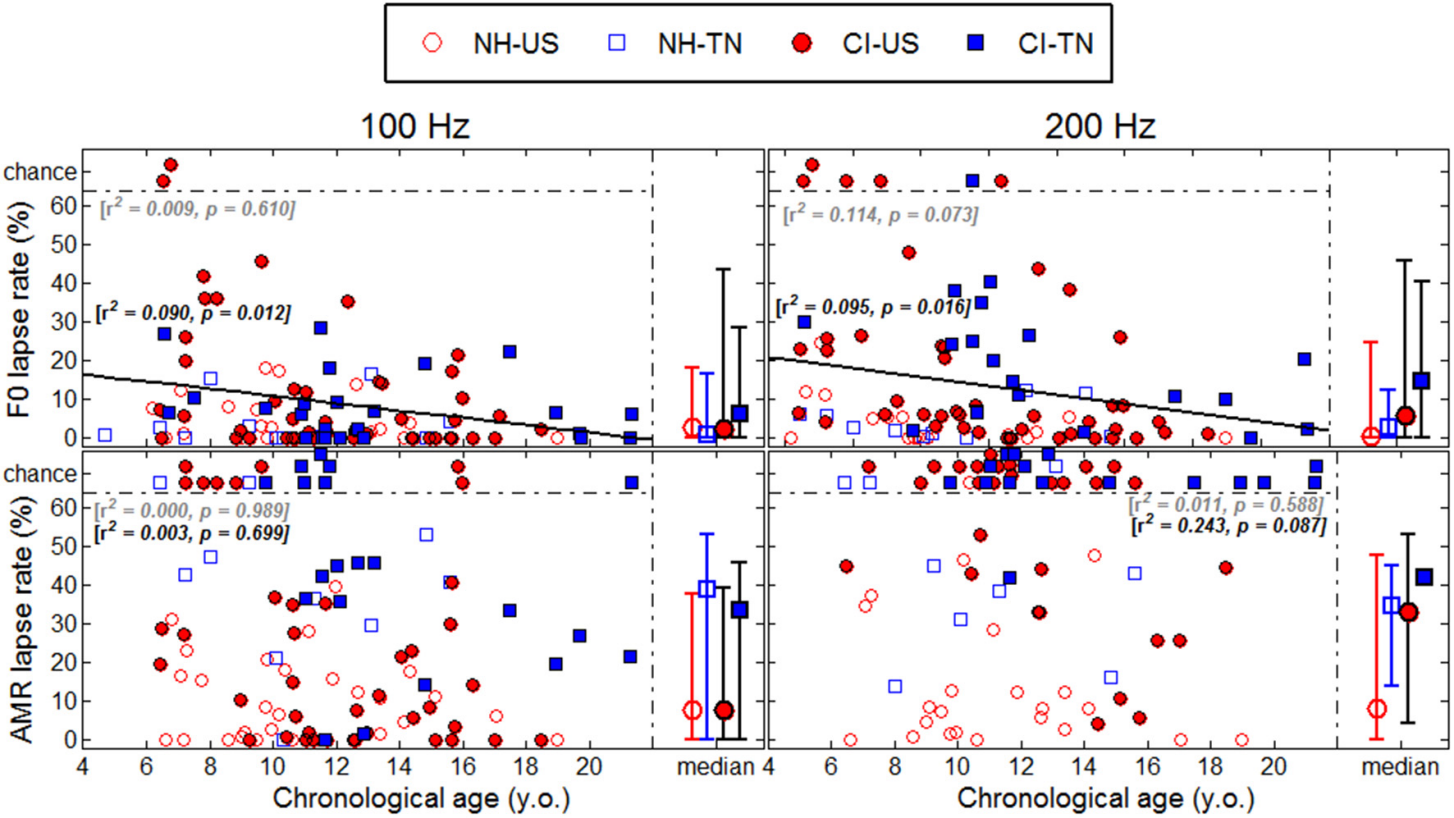
**Same as Figure 2 for lapse rates**.

Each psychophysical parameter (threshold, slope, and lapse rate) was plotted in Figures 2**–4** as a function of chronological age of all subjects. Empty symbols relate to the NH population, and filled symbols to the CI population. Red symbols relate to English-speaking children, and blue symbols to Mandarin-speaking children. For each parameter, the top panels relate to F0 discrimination, the bottom panels to AMR discrimination; the left panels relate to 100 Hz, and the right panels to 200 Hz. Homogeneity of variance between groups was fulfilled for lapse rate. This was, by far, not the case for threshold and slope when displayed on a linear scale of semitones, reflecting that sensitivity to pitch varies extremely logarithmically (i.e., logarithmically on a scale that is itself logarithmic). For this reason, an analysis of variance with two between-subject factors (hearing status × native language) was performed on the logarithm (log10) of threshold and slope, but on a linear scale for lapse rate, and the results are reported in Table 2. Listeners who could not perform a given task are reported as “chance” in the different figures (with some vertical offsets for clarity among overlapping points) and were excluded from the analysis of variance or any subsequent regression analysis.

**Table 2:** **Results of statistical analyses of each psychophysical parameter, in each of the four tasks**.

		**F0 100 Hz**	**F0 200 Hz**	**AMR 100 Hz**	**AMR 200 Hz**
Threshold	Hearing status	*F*_(1, 95)_ = 414.7, *p* = 0.00	*F*_(1, 85)_ = 341.0, *p* = 0.00	*F*_(1, 78)_ = 2.8, *p* = 0.10	*F*_(1, 38)_ = 7.7, *p* = 0.01
	Language	*F*_(1, 95)_ = 0.1, *p* = 0.79	*F*_(1, 85)_ = 0.4, *p* = 0.52	*F*_(1, 78)_ = 2.9, *p* = 0.09	*F*_(1, 38)_ = 3.0, *p* = 0.09
	Interaction	*F*_(1, 95)_ = 0.0, *p* = 0.97	*F*_(1, 85)_ = 0.7, *p* = 0.39	*F*_(1, 78)_ = 0.1, *p* = 0.74	*F*_(1, 38)_ = 4.1, *p* = 0.05
Slope	Hearing status	*F*_(1, 95)_ = 310.3, *p* = 0.00	*F*_(1, 85)_ = 235.5, *p* = 0.00	*F*_(1, 78)_ = 2.1, *p* = 0.15	*F*_(1, 38)_ = 9.6, *p* = 0.00
	Language	*F*_(1, 95)_ = 2.8, *p* = 0.10	*F*_(1, 85)_ = 0.0, *p* = 0.89	*F*_(1, 78)_ = 0.0, *p* = 0.95	*F*_(1, 38)_ = 5.7, *p* = 0.02
	Interaction	*F*_(1, 95)_ = 0.3, *p* = 0.58	*F*_(1, 85)_ = 0.6, *p* = 0.44	*F*_(1, 78)_ = 0.7, *p* = 0.41	*F*_(1, 38)_ = 6.2, *p* = 0.02
Lapse rate	Hearing status	*F*_(1, 95)_ = 2.8, *p* = 0.10	*F*_(1, 85)_ = 13.2, *p* = 0.00	*F*_(1, 78)_ = 0.2, *p* = 0.63	*F*_(1, 38)_ = 2.7, *p* = 0.11
	Language	*F*_(1, 95)_ = 0.3, *p* = 0.60	*F*_(1, 85)_ = 2.0, *p* = 0.16	*F*_(1, 78)_ = 31.7, *p* = 0.00	*F*_(1, 38)_ = 2.9, *p* = 0.09
	Interaction	*F*_(1, 95)_ = 0.0, *p* = 0.95	*F*_(1, 85)_ = 1.3, *p* = 0.26	*F*_(1, 78)_ = 1.2, *p* = 0.28	*F*_(1, 38)_ = 0.2, *p* = 0.68

### Sensitivity to F0

NH children had a very fine sensitivity to F0, whereas CI children performed more poorly. The statistics reported in Table 2 revealed a very consistent pattern across the three psychophysical parameters. There was a main effect of hearing status, but no main effect of native language and no interaction. These results provide strong evidence that speaking a tonal language has no influence on the sensitivity to F0. The CI children displayed large and consistent deficits. Thresholds were on average about 2–3 semitones for CI children against 10–20 cents for NH children (Figure 2). Slopes were also steeper for NH children than for CI children: they were on the order of 200–400% per semitone for NH children against 10–20% per semitone for CI children (Figure 3). For lapse rate, differences between NH and CI children were not as obvious, but there was a large variability in lapse rate within the CI population at both 100 and 200 Hz (Figure 4). Two young children could not perform the tasks at either F0: one had been implanted only 4 months prior to test (see Section Duration of CI Experience) and the other relied heavily on a contralateral hearing aid for communication (leaving her with a relatively poor hearing once the hearing aid was removed during the experiment). Four other CI children could only perform the task at 100 Hz, but not at 200 Hz. Performance in the sensitivity to F0 was thus extremely variable among implanted children.

Age effects were of particular interest in this study. Each of the psychophysical parameters was passed through a regression analysis (again on the log-transform of the data, except for lapse rate). Because native language had no effect, the regression was performed on the combined data across the two linguistic backgrounds, and separated into the two groups, NH (continuous black lines) or CI (interrupted gray lines). Only those that revealed a significant correlation were reported as straight lines. Correlations observed with chronological age in the implanted children were consistent across psychophysical parameters. Threshold decreased, slope increased, and lapse rate decreased, with chronological age. Note however that only about 10% of the variance was explained by this factor. For NH children, chronological age explained up to 27% of the variance in thresholds at a F0 of 200 Hz, but correlations did not reach significance for the other parameters. Thus, older listeners tended to perform better than younger listeners, in both populations, consistent with an earlier study on pure tones discrimination (Kopelovich et al., 2010), but there were clearly other sources of inter-subject variability (see General Discussion).

### Sensitivity to AMR

NH children performed much more poorly in the AMR discrimination task and therefore differences between NH and CI children were overall reduced. At a rate of 100 Hz, there was no effect of hearing status, no effect of native language, and no interaction between the two, for both threshold and slope. At a rate of 200 Hz, many children performed at chance level, so group samples became very unequal. Only one CI_TN and five NH_TN children could perform the task, so differences between CI_US and NH_US, were more meaningful since they were based on larger samples and these differences were not significant for threshold, slope, or lapse rate. Taken as a whole, the results of the AMR task reflected that speaking a tonal language did not greatly influence sensitivity to the pitch of a modulated temporal envelope. Differences between NH and CI children were overall small if significant, and the large number of CI children who could not perform the task at all was perhaps the strongest argument that NH children performed a little better than CI children in this task. Regression analyses with chronological age were also performed in this task, but none of the correlations were significant.

### Age at implantation

**Figure 5:**
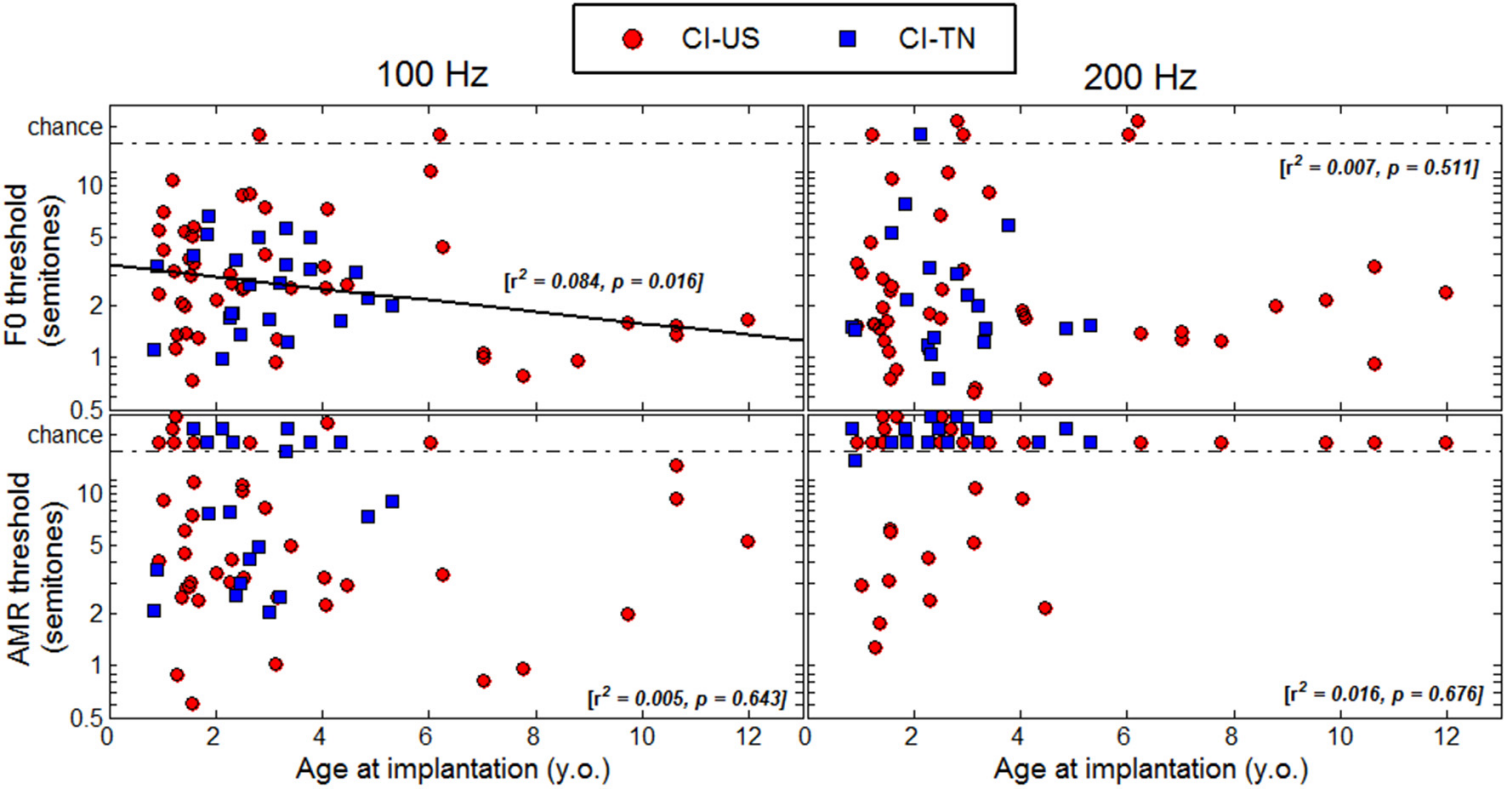
**Thresholds as a function of age at implantation**.

Figure 5 shows thresholds plotted as a function of age at implantation. Most of the CI children in the present study were implanted below 5 years of age and only eight were implanted above 7 years of age. A regression analysis (again combined across the two linguistic backgrounds) revealed only one significant correlation at a F0 of 100 Hz, and this correlation went in the opposite direction to the proposed hypothesis. This trend was largely driven by the few children implanted above 7 years of age, among which four were not profoundly deaf until 5–8 years of age. To extract this component, a multiple regression analysis was performed with (1) chronological age, (2) age at implantation, and (3) age at profound hearing loss. Note that the factor *duration of CI experience* was excluded since it is co-linear to the first two factors. The partial correlation relative to chronological age was *r* = −0.317 (*p* = 0.009), and *r* = −0.326 (*p* = 0.012), for F0s at 100 and 200 Hz. As mentioned above, about 10% of the variance was explained by this factor. For these two F0s, the partial correlation relative to age at implantation was *r* = −0.108 (*p* = 0.386), and *r* = 0.024 (*p* = 0.860), and the partial correlation relative to age at profound hearing loss was *r* = −0.180 (*p* = 0.146) and *r* = −0.065 (*p* = 0.630). Thus, there was no evidence that age at implantation or age at profound hearing loss were critical factors to F0 sensitivity. In the AMR task, none of the partial correlations were significant.

### Duration of CI experience

**Figure 6:**
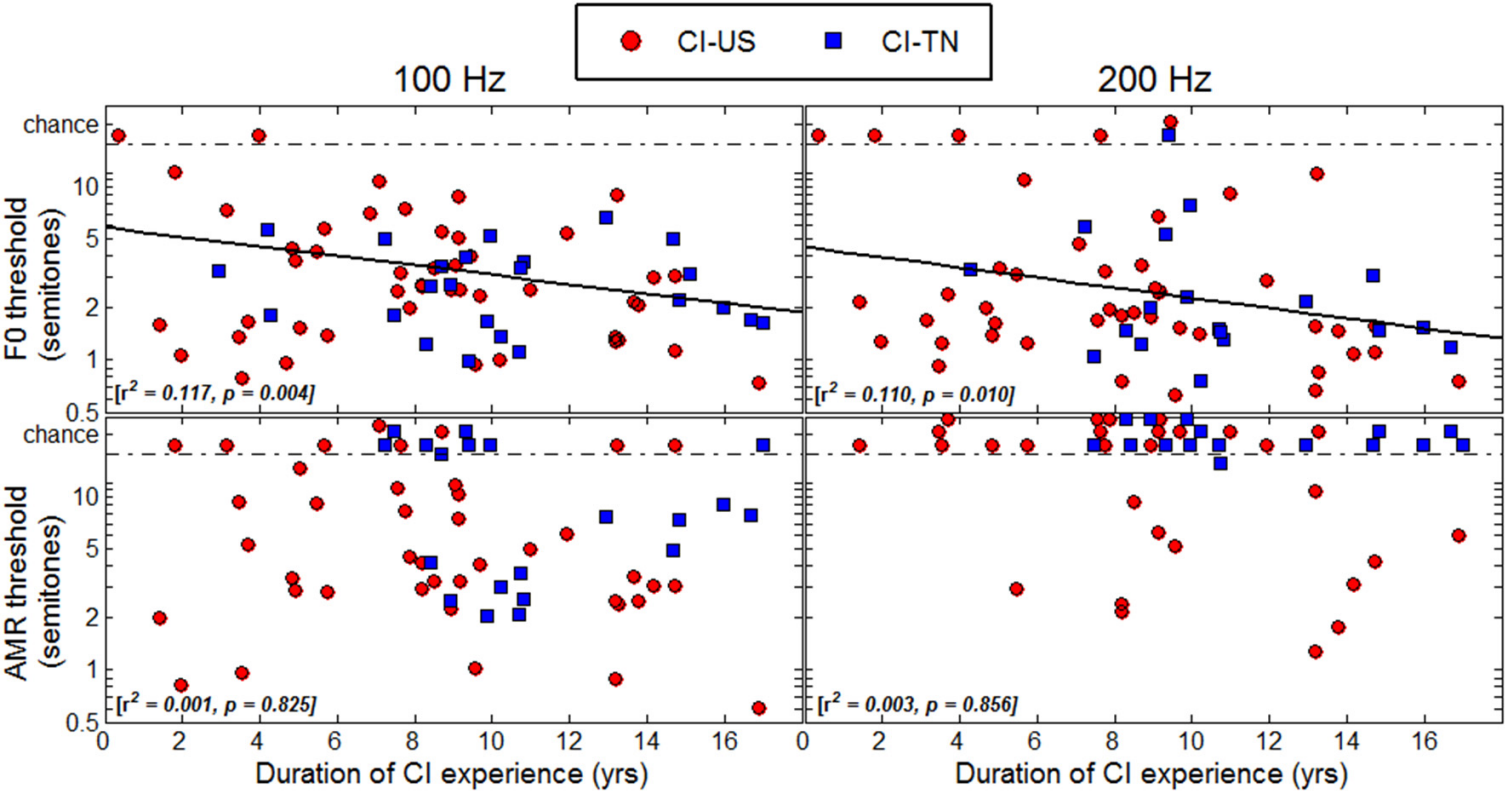
**Thresholds as a function of duration of CI experience**.

Thresholds were plotted as a function of duration of CI experience in Figure 6 to determine whether children who had longer experience with their implant would perform better than children who had less experience. A regression analysis revealed significant correlations with this variable in the F0 task at both 100 and 200 Hz, explaining about 10% of the variance. However, one should bear in mind that in this study, duration of CI experience was very much correlated with chronological age since many children were implanted early in life. The results of the multiple regression analysis mentioned above revealed that these effects were essentially explained by chronological age, not by age at implantation. Thus, the reason why listeners with the longest CI experience tended to perform better was simply that they were the oldest listeners. There were a few particular cases that deserve attention. For instance, the 6.5-year-old child who had her implant for only 4 months could not perform the task at either 100 or at 200 Hz. She may be a typical example of how duration of CI experience may matter: there is perhaps a few months post-implantation period over which children face a good deal of adaptation beyond which performance in pitch-related tasks is relatively stable, but might improve with chronological age.

### Comparisons between tasks

Additional analyses of variance were performed for each of the four groups of listeners to evaluate how performance varied as a function of the task, i.e., F0 or AMR at 100 or 200 Hz references. The sample size of a given group varied across the four tasks, because listeners did not always complete all four tasks. Consequently, the two factors (reference × pitch cue) were treated as between-subjects factors. The results are reported in Table 3. For NH_US as well as NH_TN children, there was a very consistent pattern across psychophysical parameters: the main effect of the kind of pitch cue was always significant whereas that of reference frequency (100 vs. 200 Hz) and the interactions never reached significance. For NH children therefore, sensitivity to F0 was much better than sensitivity to AMR, irrespective of the psychophysical parameter examined.

**Table 3:** **Results of statistical analyses of each psychophysical parameter, in each of the four groups of listeners**.

		**NH_US**	**NH_TN**	**CI_US**	**CI_TN**
Threshold	100/200 Hz	*F*_(1, 84)_ = 0.4, *p* = 0.51	*F*_(1, 32)_ = 1.7, *p* = 0.20	*F*_(1, 127)_ = 0.8, *p* = 0.37	*F*_(1, 53)_ = 1.6, *p* = 0.21
	F0/AMR	*F*_(1, 84)_ = 480.0, *p* = 0.00	*F*_(1, 32)_ = 412.5, *p* = 0.00	*F*_(1, 127)_ = 10.1, *p* = 0.00	*F*_(1, 53)_ = 15.5, *p* = 0.00
	Interaction	*F*_(1, 84)_ = 3.8, *p* = 0.05	*F*_(1, 32)_ = 0.4, *p* = 0.53	*F*_(1, 127)_ = 2.0, *p* = 0.16	*F*_(1, 53)_ = 5.1, *p* = 0.03
Slope	100/200 Hz	*F*_(1, 84)_ = 0.2, *p* = 0.62	*F*_(1, 32)_ = 1.5, *p* = 0.23	*F*_(1, 127)_ = 0.8, *p* = 0.36	*F*_(1, 53)_ = 4.1, *p* = 0.05
	F0/AMR	*F*_(1, 84)_ = 336.0, *p* = 0.00	*F*_(1, 32)_ = 152.7, *p* = 0.00	*F*_(1, 127)_ = 7.9, *p* = 0.01	*F*_(1, 53)_ = 10.8, *p* = 0.00
	Interaction	*F*_(1, 84)_ = 1.6, *p* = 0.21	*F*_(1, 32)_ = 0.8, *p* = 0.39	*F*_(1, 127)_ = 0.1, *p* = 0.76	*F*_(1, 53)_ = 10.4, *p* = 0.00
Lapse rate Hz	100/200	*F*_(1, 84)_ = 0.1, *p* = 0.83	*F*_(1, 32)_ = 0.1, *p* = 0.82	*F*_(1, 127)_ = 13.7, *p* = 0.00	*F*_(1, 53)_ = 3.1, *p* = 0.09
	F0/AMR	*F*_(1, 84)_ = 9.1, *p* = 0.00	*F*_(1, 32)_ = 61.8, *p* = 0.00	*F*_(1, 127)_ = 19.9, *p* = 0.00	*F*_(1, 53)_ = 11.8, *p* = 0.00
	Interaction	*F*_(1, 84)_ = 0.6, *p* = 0.45	*F*_(1, 32)_ = 0.2, *p* = 0.65	*F*_(1, 127)_ = 9.7, *p* = 0.00	*F*_(1, 53)_ = 0.1, *p* = 0.74

A similar pattern was observed to a smaller extent for CI children, who had slightly lower thresholds, steeper slopes, and lower lapse rates in the F0 task compared to the AMR task. For CI_US children, lapse rate was also higher at 200 Hz, particularly for the AMR task. For CI_TN, the small interaction between pitch cue and reference frequency was partly caused by the unequal sample sizes, particularly for the AMR task at 200 Hz. In addition, many implanted children could not complete the AMR task (particularly at 200 Hz) and those who could, had a substantial lapse rate compared to the F0 task. To sum up, the two implanted groups displayed better sensitivity to F0 than to AMR, but differences between the two tasks were reduced compared to the differences observed among NH children.

### Homogeneity between 100 and 200 Hz?

**Figure 7:**
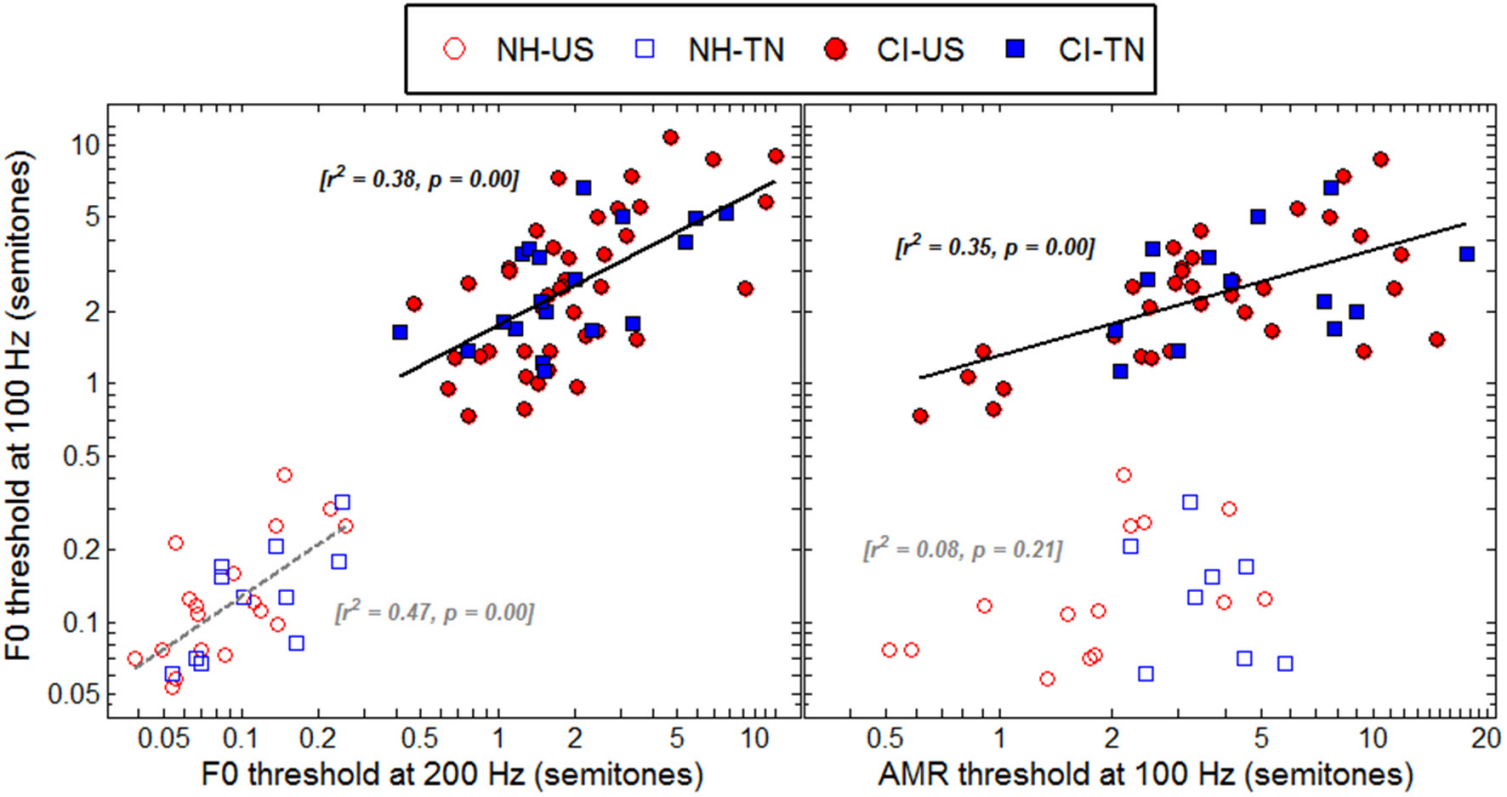
**Correlations between F0 thresholds at 100 and 200 Hz (left) and between F0 and AMR thresholds at 100 Hz (right), for all listeners who could provide data reliably above chance in these tasks**.

Within the CI population, it is fairly common to hear children reporting that they have an easier time listening to some particular voices, for instance their dad, mom, or teachers. Although sound pressure level, familiarity, articulation, and other factors may well explain those discrepancies, these reports may be grounds to hypothesize that CI children are differently sensitive to the pitch of male and female voices. For example, four CI children could perform the F0 discrimination task at 100 Hz, but not at 200 Hz. Such a pattern is consistent with the fact that at 100-Hz F0, more partials interact within a given filter band than at 200-Hz F0, and this could potentially result in a more salient envelope cue at the output of the filter. Contrary to such expectation, the median threshold was actually lower at 200 than at 100 Hz. Therefore, many children perceived pitch better at a higher F0 despite the envelopes being in principle less modulated. This sort of opposite behavior gives some support for the hypothesis that a given CI child may develop very different sensitivity at 100 and 200 Hz. On the other hand, among children who could perform the task at both F0s, this hypothesis was tested by comparing thresholds at the two F0s. As shown in the left panel of Figure 7, F0 sensitivity was correlated between 100 and 200 Hz among CI children, with an *r*^2^ of 0.38. Therefore, the present data presented somewhat conflicting evidence: a few implanted children had indeed a sensitivity to pitch restricted to the F0 range of male voices while a majority showed some homogeneity between 100 and 200 Hz. Note that, expectedly, sensitivity to F0 was very well correlated for NH children between 100 and 200 Hz, with an *r*^2^ of 0.47.

### Same or different underlying cues

For NH children, the pitch of harmonic complexes is derived primarily from periodicity in the within-channel fine structures whereas that of amplitude-modulated noise is derived from periodicity in the within-channel temporal envelopes. If these two mechanisms were genuinely distinct, sensitivity should not be strongly correlated between the F0 and AMR tasks. The right panel of Figure 7 confirms this hypothesis since there was no significant correlation among NH children between F0 and AMR thresholds at 100 Hz. In contrast, for CI children, pitch should largely be derived from periodicity in the within-channel temporal envelopes and therefore one would expect sensitivity to be more correlated between the two tasks. As shown in the right panel of Figure 7, this was indeed the case since the correlation was significant with an *r*^2^ of 0.35, just a little less than the correlation between F0 at 100 and 200 Hz.

### RT data

One classical finding in the literature on RT data is that incorrect responses are slower than correct responses when accuracy is stressed in instructions and vice-versa when speed is stressed in instructions (Swensson, 1972; Luce, 1986). The present study stressed accuracy exclusively and, as expected, RTs were overall shorter for correct than incorrect responses and, within the correct responses, RTs were also increased for the least discriminable targets (smallest differences in F0 or AMR). In both cases, the rationale is that when subjects find it difficult to know which interval contains the target, they take a little more time to make their decision. On average over all trials of all listeners, the RT was about 500-ms longer for incorrect than for correct responses, in the two F0 tasks, and this difference was about 200-ms in the AMR tasks. *T*-tests with Bonferroni corrections for multiple comparisons revealed that these differences were significant for each group in the F0 tasks, but not always significant in the AMR tasks. This result suggests that children sustained a high level of attention in the F0 tasks consistently, and behaved with precaution when they were not sure of the correct answer. This studious behavior could have disappeared in the AMR tasks for some children who experienced great difficulties and somehow lost interest in these tasks.

## General discussion

The present study showed consistent evidence that pitch sensitivity is largely impaired in CI children compared to their NH peers. NH children displayed thresholds with a median of 12.5 and 9 cents for a F0 at 100 and 200 Hz, and a median of 2.6 and 2.7 semitones for an AMR at 100 and 200 Hz. In comparison, CI children displayed thresholds with a median of 2.7 and 1.7 semitones for a F0 at 100 and 200 Hz, and a median of 3.4 and 4.3 semitones for an AMR at 100 and 200 Hz. Therefore, F0 thresholds for implanted children were on the order of magnitude of AMR thresholds for NH children, consistent with the idea that pitch was primarily derived from temporal envelopes. This was not unexpected given that the current devices used an envelope-based processing strategy. Note that the three children who used the FSP strategy did not obtain finer F0 thresholds (between 3 and 5 semitones across 100 and 200 Hz), but it is relatively difficult to know how much fine structure information did these children really receive. None of the children in our cohort used strategies such as FS4 (*Med-El*) which might have a stronger potential to convey fine structure cues. Thus, the clear deficits in pitch sensitivity that many implanted children suffer from may be strongly related to the use of envelope-based coding.

While thresholds and slopes may in theory be independent of one another, they were very consistently related in the present dataset. NH children displayed slopes with a median of 227 and 288% per semitone for a F0 at 100 and 200 Hz, and a median of 11% per semitone for an AMR at both rates. In comparison, CI children displayed slopes with a median of 10 and 16% per semitone for a F0 at 100 and 200 Hz, and a median of 8 and 9% per semitone for an AMR at 100 and 200 Hz. Therefore, F0 slopes for implanted children were on the order of magnitude of AMR slopes for NH children, leading to the same conclusion.

Since pitch is processed temporally in most current CIs, one might wonder why implanted children performed even worse in the AMR tasks than in the F0 tasks. This is likely due to even greater degradation in the representation of the noise envelope modulations through CI processors. An implant processor deals with a harmonic complex by filtering it into different spectral bands. In the most basal channels, the beating between unresolved partials results in deep envelope modulations, and with a consistent rate across channels, which are effectively transmitted to modulate trains of electrical pulses delivered to the auditory nerve. In contrast, the modulation of the noise stimuli is broadband. This means that there are interactions between the bandwidth of a given filter and the modulation of the filtered waveform. Within-channel envelopes may thus be modulated at different rates and with reduced depths, which must hinder the ability to detect modulation as well as the ability to find a common rate across channels.

In Deroche et al. (2012), using NH children only, the median threshold (also standardized at a *d*′ of 0.77) was 13 cents for the F0 task at 100 Hz, and 1.4 semitones for the AMR task at 100 Hz. The median slope was 156% per semitone for the F0 task, and 17% per semitone for the AMR task. The results obtained in the present F0 task at 100 Hz for NH children were thus consistent with the previous study [*t*_(34)_ = 0.4, *p* = 0.70 for threshold, and *t*_(34)_ = −1.6, *p* = 0.13 for slope], despite the use of different experimental setups, and the fact that sounds were played through loudspeakers instead of headphones. However, sensitivity to AMR was a little worse in the present study [*t*_(43)_ = −2.2, *p* = 0.04 for threshold], which may be due to the smaller level roving of ±1 dB in the previous study.

Loudness variations are likely to have played a detrimental role in the listeners' judgments. First, a majority of CI children performed relatively well, above 70 or 80% during training which presented all stimuli at 65 dB SPL, and yet could not perform the exact same task during the test which presented each of the three intervals with a roving of ±3 dB. Second, even NH_US children performed less well in the AMR task at 100 Hz, compared with the previous study that used only ±1 dB level roving. Therefore, both NH listeners discriminating the pitch of AM noise stimuli and CI listeners discriminating the pitch harmonic complexes display some difficulties in ignoring loudness changes. In the case of implanted children particularly, the binning of sound levels into different levels of electrical currents may have resulted in considerably large differences in loudness between intervals that may be extremely hard to ignore.

The variability in the pitch sensitivity among implanted children is similar to the large variability observed in adult CI patients in various tasks, but sources of variability might be different between post-lingually deaf adults and early-implanted children. Our analyses suggested that chronological age could explain about 10% of the variance, but once the variance from this factor was extracted, age at implantation, duration of CI experience, and age at profound hearing loss had little influence. Linguistic background did not seem to qualify either as an important account of this variability. This was quite an unexpected result, considering that pitch discrimination can be trained and improved in children, and our initial hypothesis that children developing in a tone language environment would receive continuous and natural training in pitch discrimination seemed reasonable. For instance, Kopelovich et al. (2010) developed a video game to measure pure tones discrimination at 0.5, 1, and 3 kHz, in young children with NH or CI, in free-field conditions (as well as electrode discrimination for CI children). As in this study, CI children used their standard implant settings. In line with the present results, they observed a better frequency discrimination performance for NH than for CI children, and an effect of chronological age in both populations, between 4 and 16 years old. More interestingly, they found training effects during the course of an hour, which asymptoted at a younger age in the NH than in the CI population, and did not occur for children older than about 10 years of age in both populations. These results are evidence that sensitivity to pitch (at least in the form of pure tones) can be sharpened with even little practice, but this procedural improvement reaches a plateau relatively quickly. Thus, it is in theory possible that over the age range examined in this study, English-speaking children had already “caught up” on their Mandarin-speaking peers through incidental training, but that linguistic background could result in differential pitch sensitivity at an even younger age range than tested in the present study. Alternatively, it may be that the benefits of speaking a tonal language for pitch sensitivity are specialized for the kind of pitch variations that occur in natural stimuli and might not generalize well to equal-amplitude harmonic complexes with static F0s. Barry et al. (2002) examined the ability of young children, between 3 and 11 years old, with NH or CI, to discriminate tonal contrasts in Cantonese, spoken by an arbitrary syllable. They found that CI children could discriminate several tone contrasts successfully, but had difficulties discriminating tones with relatively low F0s. They did not observe effects of chronological age, age at implantation, or duration of CI experience. Therefore, their results also suggest that plasticity-related factors play little role in a pitch-related task in CI children.

Further statistical attempts were performed to probe other potential causes of the large inter-subject variability. A between-subjects analysis of variance among the three manufactures (Cochlear, Adv. Bionics, and Med-El) did not reveal any effect [*F*_(2, 68)_ = 1.3, *p* = 0.266; *F*_(2, 59)_ = 0.3, *p* = 0.736 for the F0 thresholds at 100 and 200 Hz respectively; and *F*_(2, 45)_ = 2.2, *p* = 0.121; *F*_(1, 12)_ = 0.4, *p* = 0.534 for the AMR tasks at 100 and 200 Hz, respectively]. Gender, side of the ear tested, and number of implants (unilateral vs. bilateral) were also examined. The main effects of gender and side of the ear tested did not reach significance, nor did any interaction between these three factors [*p* > 0.097]. However, there was a main effect of number of implants: children with a single CI had lower thresholds than children implanted bilaterally at 100-Hz F0 (2.7 vs. 4.4 semitones) as well as at 200-Hz F0 (2.0 vs. 3.4 semitones) [*F*_(1, 68)_ = 5.2, *p* = 0.026 and *F*_(1, 59)_ = 4.9, *p* = 0.031, respectively], whereas this effect did not occur in the AMR task. Note though that the demographics of the two groups (unilateral vs. bilateral) differed in at least two respects: children with a single CI were on average older than children implanted bilaterally (13.0 vs. 11.1 y.o.) [*F*_(1, 70)_ = 4.5, *p* = 0.038], and they had on average a later onset of profound hearing loss than children implanted bilaterally (1.5 vs. 0.6 y.o.) [*F*_(1, 70)_ = 4.8, *p* = 0.032]. These two groups did not differ in age at first implantation [*p* = 0.285] and duration of experience with the first CI [*p* = 0.223]. Thus, the effect of number of implants might in this study not have been genuine, and could have resulted from differences in chronological age or perhaps from different degrees of hearing in the first years (if not months) of life. Overall, the main sources of the large inter-subject variability observed among implanted children in their sensitivity to pitch remain to be identified.

The fact that the side of the ear tested had no effect is somewhat intriguing considering that for NH listeners, the right lateral auditory cortex has a finer spectral resolution than the left (Hyde et al., 2008). One could thus have expected sensitivity to be finer when implanted children were tested on their left ear rather than their right ear. However, the left auditory cortex still responds to relatively large pitch changes, such as 2 semitones, and many CI children had mean thresholds beyond 2 semitones. Thus, the range of pitch differences that most CI children dealt with in the present study might have been too coarse for such an asymmetry to be observable. Alternatively, it may be that this asymmetry is less easily observable with harmonic complexes or amplitude-modulated noises than with pure tones as used in Hyde et al. (2008).

While there is clinical evidence suggesting that early implantation is beneficial to several components of language development (Fryauf-Bertschy et al., 1997; Tyler et al., 1997; Nikolopoulos et al., 1999; Kirk et al., 2002; Lesinski-Schiedat et al., 2004; Svirsky et al., 2004; Tomblin et al., 2005; Dettman et al., 2007; Holt and Svirsky, 2008; Houston et al., 2012), this result does not seem to hold for pitch sensitivity. Presumably, periodicity in the within-channel fine structures is an absolute requirement for listeners to reach sensitivity down to 10 cents. These cues being absent in most current implants, the brain has to make the best of periodicity in the temporal envelopes whose estimation from an autocorrelogram is crude (e.g., Meddis and O'Mard, 1997). Having an implant makes it hard to perceive subtle pitch differences because the cues required to achieve this fine ability are simply not delivered. Unless future developments of CIs start transmitting enough fine structure information, potential benefits of brain plasticity may be missed.

## Summary

Using a 3I-3AFC constant-stimulus procedure with a child-friendly interface, psychometric functions were measured in four groups of children, for the F0 discrimination of harmonic complexes and the rate discrimination of amplitude-modulated noise, at 100- and 200-Hz references. Children spoke English or Mandarin, and either had NH or were implanted (listening with their clinically assigned settings with envelope-based coding strategies). There were large and consistent deficits in F0 sensitivity of implanted children, compared with their NH peers, at both 100 and 200 Hz. These deficits were consistent with the fact that current implant recipients only access the periodicity of within-channel envelopes, which is known to provide much poorer discrimination abilities for NH listeners. Smaller differences were observed between the two populations in their sensitivity to AMR, but many implanted children could not perform these tasks at all. This was also not surprising, considering that the temporal envelopes are even more degraded in these cases. American and Taiwanese children performed similarly, in each population respectively, providing no support for the hypothesis that speaking a tonal language enhances pitch sensitivity. In addition, chronological age could explain about 10% of the variance in F0 thresholds among implanted children, but once this variance was extracted, factors such as age at implantation, duration of CI experience, or age at profound hearing loss did not appear to be critical. The nature of the input that the implant delivers to the brain (following current coding strategies) is poor and potential advantages of plasticity do not occur for pitch sensitivity.

### Conflict of interest statement

The authors declare that the research was conducted in the absence of any commercial or financial relationships that could be construed as a potential conflict of interest.
